# Assessment of *Methylobacterium oryzae* CBMB20 aggregates for salt tolerance and plant growth promoting characteristics for bio-inoculant development

**DOI:** 10.1186/s13568-017-0518-7

**Published:** 2017-11-21

**Authors:** Mak Chanratana, Gwang Hyun Han, Aritra Roy Choudhury, Seshadri Sundaram, Md. Abdul Halim, Ramasamy Krishnamoorthy, Yeongyeong Kang, Tongmin Sa

**Affiliations:** 1 Department of Environmental and Biological Chemistry, College of Agriculture, Life and Environment Sciences, Chungbuk National University, Cheongju, Chungbuk 361-763 Republic of Korea; 2 Indegenous and Frontiers Technology Research (IFTR) Centre, Chennai, India; 3 Tamil Nadu Agricultural University, Madurai, India

**Keywords:** *Methylobacterium oryzae* CBMB20, Salt stress, C/N ratio, Proline, PHB, EPS

## Abstract

Salinity is one of the major factors contributing to the loss of crop productivity and thereby impacting livelihood of people in more than 100 countries of the world and the area of land affected by salinity is increasing day by day. This will worsen due to various factors such as drought that might result in high soil salinity. Use of plant growth promoting rhizobacteria is one of the promising eco-friendly strategies for salinity stress management as part of sustainable agricultural practices. However, it requires selecting rhizobacteria with good survivability and adaptation to salt stress. In this study we report aggregation of *Methylobacterium oryzae* CBMB20 cells grown in media containing high C/N ratio (30:1) than in media containing low C/N ratio (7:1). Aggregated *Methylobacterium oryzae* CBMB20 cells exhibited enhanced tolerance to UV irradiation, heat, desiccation, different temperature regimes, oxidative stress, starvation and supported higher population in media. Poly-β-hydroxybutyrate accumulation, exopolysaccharide production, proline accumulation and biofilm formation were good at 100 mM salt concentration with good microbial cell hydrophobicity at both 50 and 100 mM than other concentrations. Both the aggregated and non-aggregated cells grown under 0–200 mM salt concentrations produced IAA even at 200 mM salt concentration with a peak at 100 mM concentration with aggregated cells producing significantly higher quantities. ACC deaminase activity was observed in all NaCl concentrations studied with gradual and drastic reduction in aggregated and non-aggregated cells over increased salt concentrations.

## Introduction

Soil salinity adversely affects the livelihood of people in more than 100 countries, as they occupy about 831 million ha (Mha) all over the world. Out of this area, 397 Mha (47.8%) are saline, while 434 Mha (52.2%) are sodic. On the regional scale, Asia, the Pacific and Australia together have the largest (30%) salt affected land (Sharma and Chaudhari 2012). It is claiming about 3 hectares of arable land from conventional crop farming every minute. Microbial inoculants play a vital role for alleviation of salt stress for crop growth and developments. Plant growth-promoting bacteria (or PGPB) belong to a beneficial and heterogeneous group of microorganisms found in the rhizosphere and are capable of enhancing the growth of plants and protecting them from diseases and alleviate abiotic stresses. The mechanisms by which they stimulate plant growth involve the availability of nutrients originating from genetic processes, such as biological nitrogen fixation, phosphate solubilization, stress alleviation through the modulation of ACC deaminase expression, and production of phytohormones and siderophores, among several others (Glick 2012; Souza et al. 2015).

Identification and development of stress-tolerant microbial strains associated with the roots of agronomic crops can lead to improved fertility of salt-affected soils (Maheshwari 2012). However, development of bio-inoculants for agricultural usage requires strategies that can prevent the rapid decline of inoculant populations and extend the shelf life of formulations for a longer period both under storage conditions (Bashan et al. 2014) and after inoculation into soil. It is desirable to study the physiological state of bio-inoculants (cell growth, physiological characteristics etc.), and its role in the survival of the inoculants under stress environment (Catroux et al. 2001; Kadouri et al. 2005). Various biochemical characters of bio-inoculants include sporulation (González-Pastor 2011); aggregation/floc cells (Burdman et al. 2000); accumulation of exopolysaccharide (EPS) (Bahat-Samet et al. 2004), poly-β-hydroxybuterate (PHB), and biofilm formation (Kadouri et al. 2003). These traits either individually or in combination can enhance the survival of inoculants against various stress conditions. Aggregation of inoculants has been reported to be a promising approach for large-scale production and easy harvest from the culture medium (Neyra et al. 1997). Flocculated or aggregated cells *Azospirillum* sp. has been reported to maintain high survival rate during long periods of storage, and even on the spermoplane and spermosphere of plants (Joe and Sivakumar 2009; Joe et al. 2010, 2012).

*Methylobacterium* sp. are capable of using various carbon sources including methanol, methylamine and can colonize the phyllosphere and the rhizosphere of plants (Idris et al. 2004; Omer et al. 2004; Whipps et al. 2008; Madhaiyan et al. 2009). In this study, aggregated *M. oryzae* CBMB20 grown in ammonium mineral salt (AMS) medium with high C/N ratio was studied for their endurance under various abiotic stresses. Additionally, production of proline, IAA, ACC deaminase, Poly-β-hydroxybutyrate (PHB) accumulation, exopolysaccharide (EPS) production, and biofilm formation under salt stress conditions were also evaluated.

## Materials and methods

### *Methylobacterium oryzae* CBMB20 growth in high and low C/N ratio

A modified protocol of Burdman et al. (1998) was used to make the high and low C/N ratio media. 10 ml (8.00 log CFU ml^−1^) of secondary culture of *Methylobacterium oryzae* CBMB20 (DSM 18207^T^) was transferred to sterile high C/N and low C/N medium with sodium succinate used as the sole carbon source (Madhaiyan et al. 2009). Flasks containing 100 ml of culture media was incubated in shaker at 180 rpm and maintained at 30 °C for 72 h. The bacterial suspension equivalent to 9.00 log CFU ml^−1^ was used in all subsequent experiments.

### Evaluation of growth and biochemical characteristics of cells

Estimation of the bacterial cell dry weight and cell hydrophobicity and aggregation were carried out following standard protocol described earlier (Grimaudo and Nesbitt 1997; Joe et al. 2013; Kumar et al. 2014; Perpetuini et al. 2017). To measure dry cell weight (DCW), centrifuge tubes were dried to constant weight and 10 ml of bacterial culture was harvested and washed with saline solution. The pooled samples were kept in pre-weighed centrifuge tubes and dried in the oven at 80 °C overnight. Dry weight was recorded as mg per ml of medium (mg/ml).

The adhesiveness of bacteria to xylene gave the idea about cell surface hydrophobicity or hydrophilicity, while the values obtained from the other two solvents. i.e., ethyl acetate and chloroform were regarded as a measure of acidic (electron acceptor) or basic (electron donor) characteristics of bacteria, respectively. For aggregation studies, 1 ml of bacterial culture was added to 10 ml aggregation buffer which consisted 20 mM Tris–HCl (pH 7.8), 0.01 mM MgCl_2_, 0.1 M NaCl, and 0.02% sodium azide.

Bacterial exopolysaccharide (EPS) was estimated by phenol–sulfuric acid method using glucose as standard (Albalasmeh et al. 2013). Biofilm formation was estimated following Djordjevic et al. (2002) and Merritt et al. (2005).

Poly-β-hydroxybutyrate (PHB) accumulation within the cells was analyzed by Gas Chromatograph (GC 6890 N, Agilent Inc., HP-5 column, 30 m × 320 µm × 0.25 µm) equipped with flame ionization detector (FID). Briefly, 10–20 mg of dry cells were added into 10 ml screw cap test tubes with 2 ml chloroform and 2 ml acidic methanol (2.8 M H_2_SO_4_ in methanol). One gram of benzoic acid was dissolved in 1 l methanol and used as an internal standard. Methanolysis was performed at 100 °C for 3.5 h in an oven and mixed by shaking every 30 min. After cooling at room temperature, 1 ml of distilled water was added into the tubes; the mixture was shaken vigorously for 1 min and kept for about 30 min for phase separation. One microliter of the sample was injected and nitrogen (99.99%) was used as carrier gas with a constant flow rate of 20 ml/min. The GC separation was performed under following conditions: oven temperature programmed to increase from 60 °C (5 min) to 180 °C (5 min) at a heating rate of 4 °C/min. The injector and detector temperatures were kept at 230 and 280 °C, respectively (Kumar et al. 2014). All the analyses were conducted in triplicates.

### Abiotic stress endurance of *M. oryae* CBMB20

*Methylobacterium oryae* CBMB20 bacterial culture grown under high C/N and Low C/N medium were harvested at 72 h and washed twice with saline solution and were subsequently used in all the following experiments. All tests were performed in triplicates.

Physical stress was applied by irradiation with ultraviolet light. Ultraviolet irradiation resistance, survivability under desiccation and the starvation experiment with different salt concentrations (0, 50, 100, 150 and 200 mM NaCl) were performed pursuant to (Kadouri et al. 2003; Zhao et al. 2007; Wang et al. 2009), respectively.

Resistance to ultraviolet irradiation was tested by placing 20 ml of cells in Petri dishes and exposing them to short wavelength UV light (254 nm) using the ultraviolet lamp for 120 s (Zhao et al. 2007). Survival of cells under desiccation was tested by placing 100 µl of bacteria on slides. The slides were air dried in a laminar flow at a constant air flow of 0.45 m/s for 60 min. The slides were kept at 30 °C for 30–180 min after desiccation under laminar flow. Later the cells were suspended in potassium phosphate buffer (0.06 M, pH 6.8) and plated on nutrient agar (Kadouri et al. 2003). The starvation experiment was done by incubating 50 ml of bacterial culture in M9 medium without carbon source on a shaker at 180 rpm for 30 days. Bacterial population (cfu ml^−1^) was subsequently determined every 5-day interval (Wang et al. 2009).

Aliquots of bacterial culture (100 µl) were evenly spread on 90 mm Petri dishes containing AMS agar. Simultaneously, 25 µl of H_2_O_2_ (3.0%) was placed on 13 mm Whatman discs and left to air dry for 15 min. A single disc was placed in each petri dish and incubated at 30 °C for 72 h. The size of the halo zone around the disc was taken as a measure of the bacterial sensitivity against H_2_O_2_ (Kadouri et al. 2003).

For heat stress, bacterial cells (1 ml in centrifuge tube) were incubated in a water bath at different temperatures (30, 40, 45, 48 and 50 °C) for 10 min. For cold stress, cells were incubated at 4 °C and taken out every 24 h till 192 h and the viability was studied by plating (Zhao et al. 2007).

### Cell viability of *M. oryzae* CBMB20 in glycerol during storage

Bacterial cells were harvested by centrifugation at 10,000×*g* at 4 °C for 5 min, then washed twice with saline solution and adjusted to a final concentration of bacteria (9.00 log CFU ml^−1^) in 2% (v/v) glycerol solution. The liquid form of inoculants was incubated at 4 °C, 30 °C, 40 °C and 50 °C for 3 months. One milliliter sample was withdrawn at specific time intervals (30 days), and the number of viable cells were estimated by the plate count method (CFU ml^−1^) in a series of dilution (in 0.85% saline) and then plated on a nutrient agar plate.

### Evlauation of *M. oryzae* CBMB20 under salt stress

The sensitivity of cells to osmotic stress was determined by exposing the bacterial cells to 50, 100, 150 and 200 mM of NaCl on a shaker at 180 rpm for 72 h and incubating them at 30 °C (Zhao et al. 2007). Free proline accumulation was determined as described earlier (Sandhya et al. 2010). Briefly, 10 ml of bacterial suspension was harvested by centrifugation at 3000×*g* for 5 min. The cell pellet was suspended in 70% ethanol and boiled at 60 °C in a water bath for 45 min. The suspension was centrifuged at 10,000×*g* for 15 min and the supernatant was estimated for proline content (Bates et al. 1973; Mahipant et al. 2017). Plant growth promoting characteristics like production of indole-3 acetic acid (IAA) in the presence and absence of tryptophan (Bano and Musarrat 2003) and 1-aminocyclopropane-1-carboxylate (ACC) deaminase activity by growing the bacteria on DF minimal salt medium (Li et al. 2011) amended with 3 mM ACC as nitrogen source (Penrose and Glick 2003) with different concentrations of salt (0, 50, 100, 150 and 200 mM NaCl) were also studied.

### Statistical analysis

All data were normalized and subjected to analysis of variance (ANOVA). Significant differences among means were tested with Duncan’s Multiple Range Test (DMRT) at P < 0.05 using SAS Version 9.1.3 service pack 4 (designed by SAS Institute Inc., Cary, North Carolina, USA) for all data in the experiments.

## Results

### Growth of *M. oryzae* CBMB20 under high and low C/N ratio

Growth and population of *M. oryzae* CBMB20 was more in high C/N ratio medium than the low C/N ratio medium (Table 1). The population increased both in terms of cfu ml^−1^ and cell dry weight compared to low C/N medium. Interestingly the bacterial cells grown in high C/N ratio medium showed aggregation of cells evidenced by the significantly higher cell aggregates. The formations of cell aggregation was visible within 24 h of culture. In consonance with the population, the poly-hydroxybutyrate (PHB) content was twofold higher in the aggregated *M. oryzae* CBMB20 cells grown in high C/N ratio medium. Similarly, the exopolysaccharide (EPS) analyzed was also threefold higher. Consequently, the biofilm formation in aggregated cells registered 35% enhancement compared to their non-aggregated counterparts grown in low C/N media.

**Table 1:** Cell growth and physiological characteristics of aggregated and non-aggregated *M. oryzae* CBMB20 cells

Treatment	Population^a^	Dry weight^b^	PHB production^c^	EPS production^d^	Biofilm formation^e^	Aggregation^f^	Hydrophobicity^f^
High C/N	8.48 ± 0.028^a^	6.51 ± 0.22^a^	0.53 ± 0.26^a^	0.64 ± 0.04^a^	0.294 ± 0.0^a^	35.96 ± 0.33^a^	40.02 ± 0.27^a^
Low C/N	7.92 ± 0.027^b^	3.45 ± 0.11^b^	0.23 ± 0.40^b^	0.20 ± 0.02^b^	0.189 ± 0.0^b^	22.37 ± 3.16^a^	11.65 ± 0.44^b^

^a^log CFU ml^−1^

^b^mg ml^−1^

^c^mg PHB g^−1^ cell dry weight

^d^mg EPS g^−1^ cell dry weight

^e^Amount of dye (crystal violet) used to stain was quantified by measuring at OD_595nm_

^f^Percentage (%)

### Stress endurance of *M. oryzae* CBMB20

**Fig. 1:**
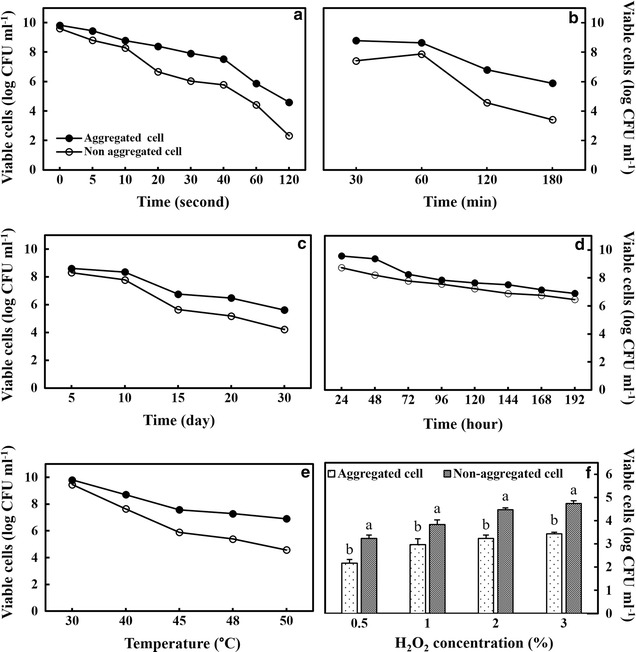
The impact of various stresses on the growth of *M. oryzae* CBMB20. **a** UV irradiation. **b** Desiccation. **c** Starvation and **d** cold (4 °C). **e** Heat (30–50 °C), **f** oxidative (H_2_O_2_). All data represents mean ± SE of three replicates and differences between means analyzed using DMRT test (*P* ≤ 0.05)

Exposure to different stress conditions like UV, desiccation and starvation stress revealed that aggregated *M. oryzae* CBMB20 was more tolerant than non-aggregated cells (Fig. 1). When bacteria were exposed to short UV wavelength at 254 nm, the population of non-aggregated of bacterial cells dramatically decreased to less than 20% survival within 120 s of UV treatment (Fig. 1). Under desiccation stress, the survival rate of non-aggregated cells declined to 30% compared to more than 60% survival of aggregated cells (Fig. 1). Besides that, when *M. oryzae* CBMB20 was exposed to starvation stress, aggregated cells showed higher survival than non-aggregated cells (Fig. 1).

**Fig. 2:**
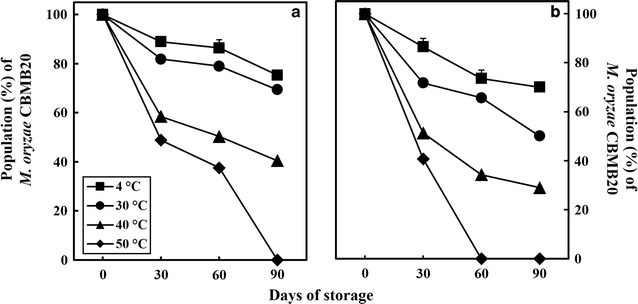
The impact of a range of temperature on the survival of **a** aggregated and **b** non-aggregated cells of *M. oryzae* CBMB20. All data represents mean ± SE of three replicates and differences between means analyzed using DMRT test (*P* ≤ 0.05)

When incubated at different temperatures, the aggregated cells survived better at 4, 30 and 40 °C after 3 months of storage compared to non-aggregated cells (Fig. 2). However, further raising the temperature to 50 °C showed a slow but gradual and uniform decline in survival of both aggregated as well as non-aggregated cells. It was also observed that the difference becomes distinct among both the physiological forms immediately after 5 days of incubation (Fig. 2). However, storage at 4 °C over 192 h did not show major difference among the both the forms.

Aliquots of bacterial culture (100 µl) were evenly spread on 90-mm Petri dishes containing AMS agar. Simultaneously, 25 µl of H_2_O_2_ (3.0%) was placed on 13-mm Whatman discs and left to air dry for 15 min. A single disc was placed in each petri dish and incubated at 30 °C for 72 h. The size of the halo zone around the disc was taken as a measure of the bacterial sensitivity against H_2_O_2_ (Kadouri et al. 2003). When their growth was studied in the presence of H_2_O_2_, the non-aggregated cells were more sensitive to 0.5–3.0% H_2_O_2_ than aggregated cells that showed around 22–33% tolerance to the increasing concentrations of H_2_O_2_ (Fig. 1).

### Effect of salt stress on *M. oryzae* CBMB20

**Fig. 3:**
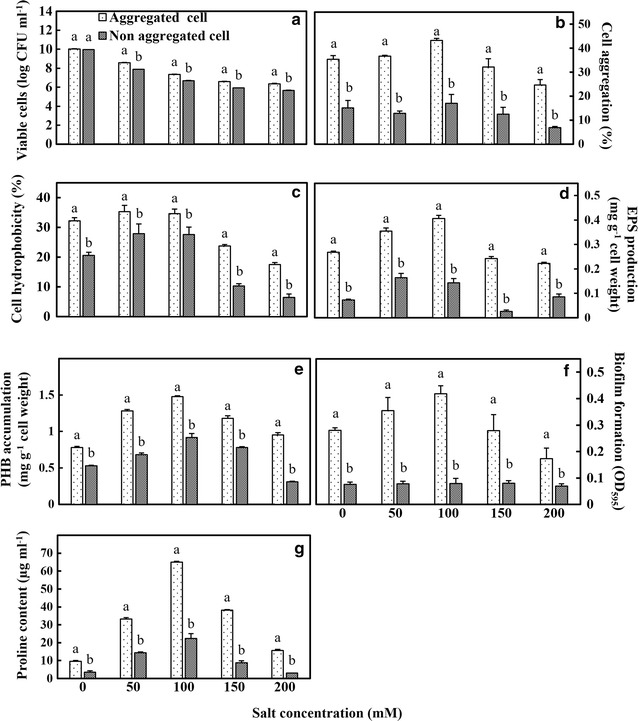
The impact of different salt concentration on *M. oryzae* CBMB20 cells. **a** Survival. **b** Cell aggregation. **c** Cell hydrophobicity. **d** EPS production. **e** PHB accumulation. **f** Biofilm formation and **g** Proline accumulation. All data represents mean ± SE of three replicates and differences between means analyzed using DMRT test (*P* ≤ 0.05)

Salt stress (0–200 mM of NaCl), in general, showed a negative correlation with the survival of the bacteria (Fig. 3). Here, both the forms i.e. aggregated and non-aggregated cell types showed sensitivity to higher salt concentration. While both cell types were able to grow well in optimal salt concentration at 100 mM NaCl, aggregated cells were quite resistant to salt stress than non-aggregated cells with better growth. Even with prolonged exposure to salt stress the survival was quite significantly higher in aggregated cells. This could be attributed to increased EPS production, biofilm formation and proline accumulation in the aggregated cells (Fig. 3d–f). While the aggregated cells continued to show cell aggregation, higher cell hydrophobicity, EPS production, biofilm formation, PHB content and proline content in cells at 50 and 100 mM salinity, the values declined at 150 and 200 mM NaCl. Compared to non-aggregated cells, up to two to threefolds EPS production was observed with increasing salt concentration till 100 mM NaCl.

**Fig. 4:**
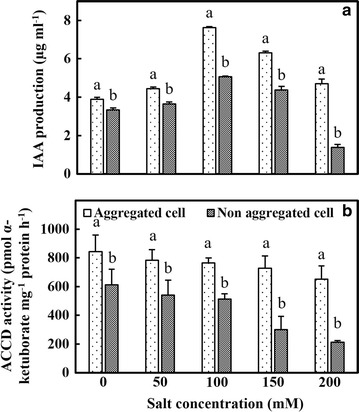
The impact of different salt concentration on production of plant growth promoting traits of *M. oryzae* CBMB20 cells. **a** IAA production. **b** ACC deaminase production. All data represents mean ± SE of three replicates and differences between means analyzed using DMRT test (*P* ≤ 0.05) All data represents mean ± SE of three replicates and differences between means analyzed using DMRT test (*P* ≤ 0.05)

When the both forms were studied for plant growth promotion traits, IAA production was higher in aggregated cells up to 100 mM salinity. Though the trend was similar in non-aggregated cells, at 200 mM NaCl concentration the IAA levels declined drastically (Fig. 4). The ACC deaminase activity was high in all the salt concentrations studied with aggregated cells showing 1.35 to 3.7-folds higher ACC deaminase activity than non-aggregated cells (Fig. 4).

## Discussion

In the present study we studied the aggregation behavior of *M. oryzae* CBMB20 grown in high C/N ratio media. Many studies have reported the formation of aggregates and flocs can potentially be utilized in the development of formulations for various agricultural practices (Neyra et al. 1997; Burdman et al. 2000; Bahat-Samet et al. 2004; Joe and Sivakumar 2009). While aggregation of cells is influenced by various chemical and physical factors, nutrient-limited conditions are well known as the major factor to induce aggregation of microorganisms (Burdman et al. 1998, 2000; Joe and Sivakumar 2009; Rathi et al. 2015).

With nutrient limited conditions mimicking the soil rhizosphere where carbon is rich and nitrogen is limited, soil microorganisms accumulate intracellular food reserve in the form poly hydroxybutyrates (PHB) and produce extracellular polysaccharides (EPS) that helps them survive better under stressed conditions (Kumar et al. 2016; González-García et al. 2015). Studies also suggest that increased extracellular and capsular polysaccharides production facilitate aggregation of *A. brasilense* cells in high C/N ratio medium (Burdman et al. 1998, 2000; Bahat-Samet et al. 2004). The aggregation observed in this study is also linked to the higher EPS production by the aggregated cells. However, the aggregation seems to be a time-dependent mechanism that occurs in cells having a low metabolic activity which is also accompanied by higher hydrophobicity (Bahat-Samet et al. 2004; Joe et al. 2013) which was significantly higher in this study also. Similar observation was made on *M. oryzae* which exhibited a higher level of aggregation, EPS, and PHB content when grown in the modified ammonium minimal salt medium (AMS) at high C/N ratio (Woo et al. 2012).

The aggregated cells could withstand UV irradiation, heat, desiccation, different temperature regimes, H_2_O_2_ stress, and starvation with better population growth than non-aggregated cells. Along with this they also showed higher Poly-β-hydroxybutyrate (PHB) accumulation, exopolysaccharide (EPS) production, proline accumulation and biofilm formation. Ability to accumulate PHB during stress could be attributed to the tolerance at higher temperature. This cannot be ruled out in the enhanced survival of *M. oryzae* CBMB20 aggregates as they are reported to help *Azospirillum brasilense*, *Aeromonas hydrophila* 4AK4, *Sinorhizobium meliloti* and recombinant *E. coli* to overcome various physico-chemical and environmental stress conditions (Kadouri et al. 2003; Zhao et al. 2007; Ratcliff et al. 2008; Wang et al. 2009). The intracellular PHB serve as energy reservoir during prolonged storage and that may be the possible reason for differential survival of aggregated *M. oryzae* CBMB20 was found at 4 and 30 °C storage temperature. The possible mechanism behind Poly-hydroxyalkanoates (PHA) accumulation was attributed to the expression of outer membrane proteins (OMPs) under stress conditions (Schweder et al. 1996; Burdman et al. 2000; Alvarez-Ordóñez et al. 2015). In *A. brasilense* strain Sp7 and *S. meliloti* the biosynthesis and accumulation of PHB was reported to help the bacteria survive under competitive environment and further help in root colonization (Kadouri et al. 2003; Ratcliff et al. 2008).

A number of bacteria are reported to adapt to challenging environmental conditions such as osmotic stress. However, this ability to survive and thrive under such conditions depends on the ability of the individual cells to survive the initial and the gradient shock. In general, *Methylobacterium* sp. cannot grow well under salt stress conditions. Schauer et al. (2011) has conferred that growth of *Methylobacterium marchantiae* is reduced by 50% when grown in 0.5% NaCl in R2A agar. Similar results were shown when *Methylobacterium pseudosasicole* was grown under NaCl concentrations of 0–7% (Madhaiyan and Poonguzhali 2014). While *M. oryzae* CBMB20 could withstand NaCl stress, its performance varied according to the NaCl concentration used. In an earlier study, two methylotrophs viz. *Methylophilus* sp. and *Methylobacterium* sp. were reported to grow in the presence of NaCl with *Methylobacterium* showing increased enzyme activity even at 500 mM NaCl concentration (Giri et al. 2013). Performance of aggregated and non-aggregated cells in this study even at 200 mM concentration substantiates the ability of *Methylobacterium* sp. to withstand higher salt concentrations. Increase in the extracellular polysaccharide (EPS) production up to two to threefolds with an increasing salt concentration till 100 mM NaCl further strengthens the ability of microbes to produce exopolysaccharides under salt stress. (Lloret et al. 1998). Similar observations were made previously in *Rhizobia* sp. (Arora et al. 2006) and *P. putida* CZ1 capable of producing EPS and biofilm providing a hydrated microenvironment to protect the cells from osmotic stress (Lin et al. 2014). Besides this, increase in Proline, the beneficial amino acid, that act as an intracellular osmolyte and protect cells (Paleg et al. 1984) from changing salt concentration up to 100 mM salt concentration observed in this study was also reported in *B. megaterium and Pseudomonas putida* (Marulanda et al. 2009), *Escherichia coli*, *Pseudomonas aeruginosa* and *Salmonella typhimurium* (Baich 1969; Csonka 1981; Krishna et al. 1979). The differences in Proline accumulation was attributed to the different abilities of the bacterial isolates under osmotic stress conditions (Marulanda et al. 2009).

Production of auxins, an important hormone that activates plant cells division, is widespread among plant growth promoting bacteria and *M. oryzae* CBMB20 is no exception to this. The aggregated cells produced IAA up to 100 mM salinity with a decline at higher concentrations. Though the trend was similar in non-aggregated cells, at 200 mM NaCl concentration the IAA levels declined drastically indicating the inherent potential of the strain to produce IAA even under stress and the protection offered by the bacterial cell metabolism at high salt stress conditions to continue to produce IAA at higher quantities. The ACC deaminase activity was high in all the salt concentrations studied with aggregated cells registering 1.35- to 3.7-folds higher ACC deaminase activity than non-aggregated cells. Earlier studies also show a number of microbial strains like *Alcaligens* sp., *Bacillus* sp., *Brachybacterium paraconglomeratum, Ochrobactrum* sp., *Pseudomonas* sp. and *Serratia* sp. to produce IAA and ACC deamilnase under salt stress (Barnawal et al. 2016; Bal et al. 2013; Glick 2014; Nakbanpote et al. 2014; Marulanda et al. 2009). Upon field application, IAA and ACC deaminase produced by bacteria were reported to stimulate root growth in a coordinated fashion (Glick et al. 2007). As the isolate, *M. oryzae* CBMB20 has the ability to produce both IAA and ACC deaminase the strain could be a potential candidate for further formulation studies both in aggregated and non-aggregated form and use in salt stressed environments.

Hence, use of plant growth promoting rhizobacteria (PGPR) is a promising low cost eco-friendly strategies for mitigating salinity stress management in sustainable agriculture. However, characterizing bacteria for performance against stress would help scientists to understand their behavior for large scale applications. The plant growth promoting bacteria, *M. oryzae* CBMB20 isolated as endophyte of rice plants could form aggregates in media containing high C/N ratio (30:1) and the aggregates grew well and withstand UV irradiation, heat, desiccation, different temperature regimes, H_2_O_2_, starvation and grow well in media. The bacteria also produced poly-β-hydroxybuterate (PHB), exopolysaccharide (EPS), and accumulate proline and form biofilms at 100 mM salt concentration with good microbial cell hydrophobicity. The ability of aggregated form to produce plant growth promoting hormones under salt stress paves way for its use in preparation of polymer based formulation for application in saline agriculture.

## Abbreviations

EPS: exopolysaccharide
PHB: poly-β-hydroxybuterate
ACC: 1-aminocyclopropane-1-carboxylate
